# The role of HGF/MET and FGF/FGFR in fibroblast-derived growth stimulation and lapatinib-resistance of esophageal squamous cell carcinoma

**DOI:** 10.1186/s12885-015-1065-8

**Published:** 2015-02-25

**Authors:** Shin Saito, Kazue Morishima, Takashi Ui, Hiroko Hoshino, Daisuke Matsubara, Shumpei Ishikawa, Hiroyuki Aburatani, Masashi Fukayama, Yoshinori Hosoya, Naohiro Sata, Alan K Lefor, Yoshikazu Yasuda, Toshiro Niki

**Affiliations:** 1 Department of Surgery, Jichi Medical University, 3311-1 Yakushiji, Shimotsuke-City, Tochigi 329-0498 Japan; 2 Department of Integrative Pathology, Jichi Medical University, 3311-1 Yakushiji, Shimotsuke-City, Tochigi 329-0498 Japan; 3 Department of Genomic Pathology, Medical Research Institute, Tokyo Medical and Dental University, 1-5-45 Yushima, Bunkyo, Tokyo 113-0034 Japan; 4 Division of Genome Science, Research Center for Advanced Science and Technology, University of Tokyo, 4-6-1 Komaba, Meguro, Tokyo, 153-8904 Japan; 5 Department of Pathology, Graduate School of Medicine, University of Tokyo, 7-3-1 Hongo, Bunkyo, Tokyo 113-0033 Japan

**Keywords:** Esophageal squamous-cell carcinoma, Stromal fibroblasts, HGF, FGFs, Lapatinib, Chemo-resistance

## Abstract

**Background:**

Although advanced esophageal squamous-cell carcinoma (ESCC) is treated using a multidisciplinary approach, outcomes remain unsatisfactory. The microenvironment of cancer cells has recently been shown to strongly influence the biologic properties of malignancies. We explored the effect of supernatant from esophageal fibroblasts on the cell growth and chemo-resistance of ESCC cell lines.

**Methods:**

We used 22 ESCC cell lines, isolated primary human esophageal fibroblasts and immortalized fibroblasts. We first examined cell proliferation induced by fibroblast supernatant. The effect of supernatant was evaluated to determine whether paracrine signaling induced by fibroblasts can influence the proliferation of cancer cells. Next, we examined the effects of adding growth factors HGF, FGF1, FGF7, and FGF10, to the culture medium of cancer cells. These growth factors are assumed to be present in the culture supernatants of fibroblasts and may exert a paracrine effect on the proliferation of cancer cells. We also examined the intrinsic role of HGF/MET and FGFs/FGFR in ESCC proliferation. In addition, we examined the inhibitory effect of lapatinib on ESCC cell lines and studied whether the fibroblast supernatants affect the inhibitory effect of lapatinib on ESCC cell proliferation. Finally, we tested whether the FGFR inhibitor PD-173074 could eliminate the rescue effect against lapatinib that was induced by fibroblast supernatants.

**Results:**

The addition of fibroblast supernatant induces cell proliferation in the majority of cell lines tested. The results of experiments to evaluate the effects of adding growth factors and kinase inhibitors suggests that the stimulating effect of fibroblasts was attributable in part to HGF/MET or FGF/FGFR. The results also indicate diversity in the degree of dependence on HGF/MET and FGF/FGFR among the cell lines. Though lapanitib at 1 μM inhibits cell proliferation by more than 50% in the majority of the ESCC cell lines, fibroblast supernatant can rescue the growth inhibition of ESCC cells. However, the rescue effect is abrogated by co-treatment with FGFR inhibitor.

**Conclusion:**

These results demonstrate that cell growth of ESCC depends on diverse receptor tyrosine kinase signaling, in both cell-autonomous and cell-non-autonomous manners. The combined inhibition of these signals may hold promise for the treatment of ESCC.

## Background

Currently, esophageal cancer is the eighth most common cancer in the world [1,2]. Esophageal cancer remains one of the least studied and most lethal malignancies [3]. Squamous-cell carcinoma accounts for 92.5% of all primary esophageal tumors in Japan and other Asian countries [4-6], while adenocarcinoma is the most prevalent histologic type of esophageal cancer in western countries [7]. Since the overall incidence and mortality of esophageal squamous-cell carcinoma (ESCC) is lower than other cancers such as breast cancer, colorectal cancer and lung cancer in western countries [8], biological studies of ESCC have been lagging behind. Advanced ESCC is treated using a multidisciplinary approach, including surgery, chemotherapy, and radiotherapy, but outcomes remain unsatisfactory [9-12].

Cancers are the end-product of accumulated effects of many genetic alterations, and the specific combination of changes is reflected in the unique characteristics of each tumor. The microenvironment of cancer cells has recently been shown to strongly influence the biologic properties of cancer [13].

A tumor consists of a dynamic mixture of tumor cells, fibroblasts, endothelial cells, immune cells and extracellular matrix. In many solid tumors, the stroma has been recognized to be important in promoting tumor proliferation, invasion, metastasis, and chemo-resistance [14,15]. The proliferation of fibroblasts is frequently seen in the invasive portion of a malignant tumor and tumors with significant proliferation of those cells are associated with a poor prognosis in colorectal cancers, breast cancers and lung cancers [16-18]. In ESCC, previous reports described that stromal fibroblasts have an important role in angiogenesis [19] and tumor differentiation [20]. Fibroblasts are associated with cancer cells at all stages of cancer progression, and their production of growth factors, chemokines and extracellular matrix facilitates the angiogenic recruitment of endothelial cells and pericytes [21].

Hepatocyte growth factor (HGF) regulates cell growth, cell motility, and morphogenesis by activating a tyrosine kinase signaling cascade after binding to the c-Met receptor [22]. HGF is secreted by mesenchymal cells including fibroblasts and promotes invasion of ESCC cells [23].

Fibroblast Growth Factors (FGFs) have been implicated in the regulation of cell differentiation, proliferation, migration and survival in many different cell types [24]. The biological activities of FGFs are mediated by FGF receptors (FGFR). FGFR2 has two different isoforms that are designated FGFR2 IIIb and FGFR2 IIIc; the former is particularly localized in epithelial cells with growth induced by FGF-1, 3, 7, 10 and the latter binds FGF-1, 2, 4, 6, 9 and is expressed mainly in mesenchymal cells [25,26]. FGFR2 positive tumor fibroblasts may provide cancer cells with a suitable microenvironment to promote cancer development and progression [27].

Lapatinib is a dual tyrosine kinase inhibitor of epidermal growth factor receptor (EGFR) and human EGFR-2 (HER2) tyrosine kinase domains [28,29]. Recently lapatinib has been evaluated for the treatment of not only breast cancer [30,31] but also gastric cancer [32] and ESCC [33].

To date, however, few studies have evaluated the role of stromal fibroblasts in ESCC. In this study, we focused on the relationship between ESCC cells and fibroblasts, the main component of cancer stroma. We prepared in vitro experimental systems to evaluate the interactions between ESCC and fibroblasts and clarify the mechanisms by which fibroblasts control proliferation of ESCC cells at the molecular level. We also studied the effects of various tyrosine kinase inhibitors to gain insight into new treatment strategies for ESCC.

## Methods

### Reagents

PHA-665752 and PD-173074 were obtained from Tocris Bioscience (Bristol, UK). Lapatinib was obtained from Bio Vision (Milpitas, USA). Recombinant human HGF, recombinant human FGF1, 7, and 10 were obtained from R & D System (Minneapolis, USA). Stock solutions of PHA-665752, PD-173074 and lapatinib were prepared in dimethyl sulfoxide and stored at −80°C until use. Stock solutions of HGF and FGFs were prepared in phosphate-buffered saline (PBS) and stored at −80°C until use.

### Esophageal squamous cell lines

We used 22 esophageal squamous cell lines: TE-1, TE-4, TE-5, TE-6, TE-8 TE-9, TE-10, TE-11, TE-14, TE-15, and EC-GI-10 were obtained from RIKEN Cell Bank (Tsukuba, Japan), while KYSE30, KYSE50, KYSE70, KYSE140, KYSE150, KYSE170, KYSE180, KYSE220, KYSE270, T.T, and TTN were obtained from Health Science Research Resources Bank (Osaka, Japan). All cancer cell lines were maintained in RPMI 1640 supplemented with 10% fetal bovine serum (FBS, Autogen Bioclear), glutamine, 100units/ml penicillin, and 100 μg/ml streptomycin, in a humidified atmosphere with 5% CO_2_.

### Fibroblasts

Primary human esophageal fibroblasts designated as HEF75 [34], HEF2111, HEF1173 and cancer associated fibroblasts designated as HECAF2111 were isolated from human esophagus tissues which were resected in the Department of Surgery, Jichi Medical University Hospital. The patient from whom the tissue was obtained had not received neoadjuvant chemotherapy or radiation therapy. The study was approved by the Jichi Medical University Ethics Committee and written informed consent was obtained from the patient.

To isolate fibroblasts [35,36], epithelial tissue was washed twice in PBS and cut into 1–2 mm^3^ pieces. Several pieces were placed in a six-well plate, and the explants cultured for 48 hours in DMEM (Dulbecco’s modified Eagle’s medium; Invitrogen) supplemented with 10% FBS, antibiotics, and glutamine at 37°C in a humidified atmosphere with 5% CO_2_. After removing the explants and non-adherent cells, the remaining cells were incubated for 1–2 weeks. The adherent cells were then trypsinized and passaged into a new culture flask at a ratio of 1:3 for further expansion. The cells were used for subsequent experimental study after the third passage.

Human lung-derived fibroblasts, HFL-III, obtained from RIKEN Cell Bank, were also used in some experiments.

### Immortalization of esophageal fibroblasts

Immortalized fibroblasts, designated as HFE75-hTERT (human telomerase reverse transcriptase), were described previously [34]. In brief, in accordance with the protocol of Lipofectamin 2000 Reagent (Invitrogen Co., Ltd.), plasmid DNA encoding hTERT (pCLXSN-hTERT), a kind gift from Dr. T. Kiyono, National Cancer Research Institute, Japan [37], and pVSV-G were co-transfected into the GP2-293 cell line. After 48 hours, the culture supernatant including the retrovirus was added to fibroblasts to induce transduction. Selection was performed with G418 (Geneticin®, Invitrogen) 48 hours after transduction. In this study, cells between the 20^th^ and 25^th^ passage after viral transduction were used. Immortalized human esophageal fibroblasts were designated as human esophageal fibroblast (HEF) 75-human telomerase reverse transcriptase (HEF75-hTERT).

### Antibodies

Rabbit polyclonal anti-phospho-Akt, anti-phospho-Erk antibodies (Cell Signaling Technology, Danvers, MA) and goat polyclonal anti-beta actin (Santa Cruz Biotechnology, Santa Cruz, CA) were used in this study.

### Gene expression profile

A comprehensive gene expression analysis was performed using an oligonucleotide microarray (GeneChip Human Genome U133A, Affymetrix, Santa Clara, CA) as described previously [38]. The data discussed in this publication have been deposited in NCBI’s Gene Expression Omnibus [39] and are accessible through GEO Series accession number GSE63941 (http://www.ncbi.nlm.nih.gov/geo/query/acc.cgi?acc=GSE63941).

### Western blot analysis

Cells were lysed in a lysis buffer consisting of 20 mmol/L Tris–HCl (pH7.4), 150 mmol/L NaCl, 50 mmol/L NaF, and 1 mmol/L Na_3_VO_4_ with a cocktail of proteinase inhibitors. After sonication, lysates were immersed in water at 98°C for five minutes and cleared by centrifugation. Protein concentrations were determined using the DC Protein Assay kit (BioRad). For Western blot analysis, equal amounts of protein samples were size-separated on 8% polyacrylamide gels and electroblotted onto a nitrocellulose membrane. Nonspecific binding was blocked by immersion of the membranes for 20 minutes in 5% skim milk in Tris-buffer saline at room temperature.

Membranes were washed with Tris-buffer saline buffer containing 0.1% Tween 20, incubated for one hour at room temperature with primary antibodies, washed, and then reacted with peroxidase-conjugated secondary antibodies. The antigen was detected using ECL Western Blotting Detection Reagents (Amersham) following the manufacturer’s instructions.

### Cell proliferation assay

Cell viability was measured using the Cell Counting Kit (CCK) 8 assay (Dojindo, Tokyo, Japan) according to the manufacturer’s instructions. ESCC cells (4-10×10^3^ cells) were plated in 96-well microtiter plates. After 24 hours, a supernatant of fibroblasts or growth factors (HGF, FGF1, FGF7, and FGF10), was added to the wells as appropriate.

The fibroblast supernatant was prepared by incubating HEF75 or HEF75-hTERT in DMEM containing 0.1%FBS for 12 hrs. The prepared supernatant was diluted at 1×, 1/2×, 1/10×, 1/100×, and 1/1000× with 0.1%FBS + DMEM and added to the ESCC cells. The final concentrations of the growth factors were: 20 ng/ml, 10 ng/ml, 2 ng/ml, 0.2 ng/ml and 0.02 ng/ml for HGF; 10 ng/ml, 1 ng/ml, 0.1 ng/ml, 0.01 ng/ml and 0.001 ng/ml for FGF1; 40 ng/ml, 20 ng/ml, 2 ng/ml, 0.2 ng/ml, 0.02 ng/ml for FGF7; and 20 ng/ml, 10 ng/ml, 1 ng/ml, 0.1 ng/ml and 0.01 ng/ml for FGF10.

After the addition of fibroblast supernatant or growth factors, cells were incubated for four days at 37°C. For inhibition experiments, cells were incubated in the presence of 1 μM of lapatinib (a dual inhibitor of epidermal growth factor receptor and human epidermal growth factor receptor 2), PHA-665752 (MET inhibitor), PD-173074 (FGFR inhibitor), or 0.1% of dimethyl sulfoxide (DMSO, negative control).

At the end of the four-day incubation, the absorbance of each well at 450 nm was measured with a reference at 630 nm using a BIO-RAD model 680XR microplate reader (Bio-Rad, Hercules, CA).

The percentage of cell viability was calculated by following formula:%cell viability = (mean absorbance in test wells)/ (mean absorbance in control well) ×100. Results were plotted as cell viability versus log_10_ (concentration of reagents).

To describe the blocking effect of PHA-665752 and PD-173074 on the growth promoting effect of fibroblast supernatant, a blunting index was calculated by the formula: blunting index (%) = 100-(fold change in the presence of PHA-665752 (or PD-173074) and fibroblast supernatant/fold change in the presence fibroblast supernatant alone) × 100.

### Enzyme-linked immunosorbent assay (ELISA)

Human HEF75, HEF75-hTERT, and some cancer cells in a confluent state were cultured for 24 hours in RPMI/DMEM containing 0.1%FBS, and the supernatant concentration was measured by Quantikine® human FGF 7 immunoassay or human HGF immunoassay (R&D Systems). Measurements were performed in accordance with the manufacturer’s instructions.

### Statistical analysis

Analysis of variance was used to compare mean values of continuous variables among three or more groups, and P-values were calculated. When the P-value was less than 0.05 and the null hypothesis was rejected, Dunnett’s test, a method for post hoc comparison, was used to compare the groups. The Jmp9 (SAS Institute Inc., Cary, North Carolina, USA) statistical software package was used for analysis.

## Results

### Effect of fibroblast supernatant on ESCC cell proliferation

**Figure 1:**
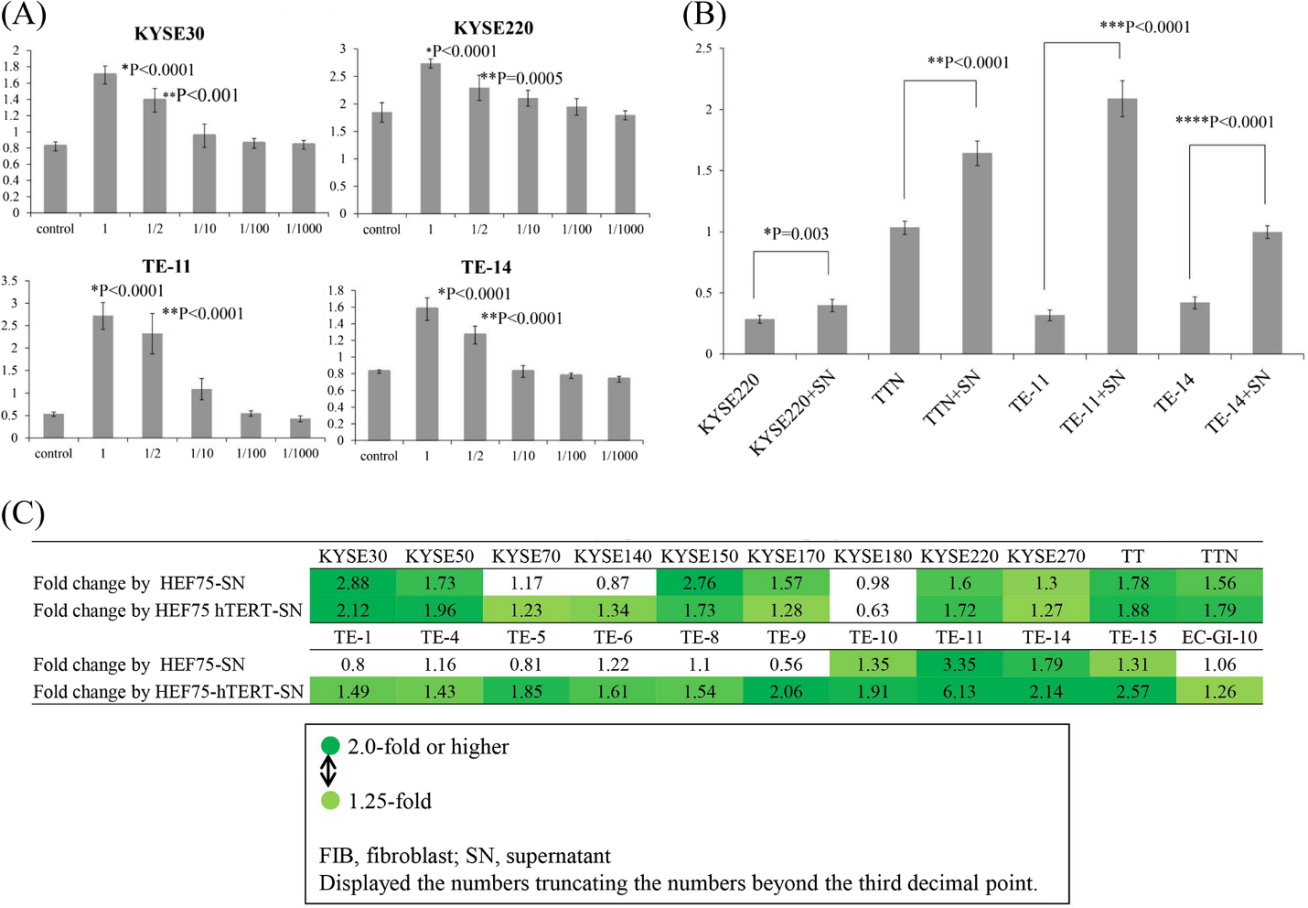
**Proliferative responses of ESCC cell lines to HEF75 and HEF75-hTERT fibroblast supernatants. (A)** KYSE30, KYSE220, TE-11, and TE-14 show significantly increased cell proliferation until a one-half dilution, as compared with the control group. For each cell line, the P- value is less than 0.001 on one-way analysis of variance. Dunnet’s test was used for comparison with the control group, and P-values were calculated. The Y axis indicates the absorbance. **(B)** Supernatants from HEF75-hTERT also significantly stimulate the proliferation of KYSE220, TTN, TE-11 and TE-14 cell lines. **(C)** Proliferative responses of 22 ESCC cell lines to fibroblast supernatants. With normal esophagus fibroblast supernatant (HEF75), 1.25-fold or higher proliferation is induced in 12 cell lines. With immortalized esophageal fibroblast (HEF75-hTERT) supernatant, 1.25-fold or higher proliferation is induced in 21 cell lines. The proliferation rate compared to controls is shown by a graduated color scale (lower panel). Fold change by HEF75 or HEF75-hTERT is the ratio of the results of the proliferation assay after addition of HEF 75 or HEF75-hTERT as compared with the control. A 1.25 fold or higher increase in proliferation as compared with control is highlighted in green. The data shown are the mean values of two or more independent experiments for each cell line.

The effect of fibroblast supernatant was evaluated to determine whether paracrine signaling induced by fibroblasts can influence the proliferation of cancer cells. Using the cell count assay, fibroblast supernatant from HEF75 and HEF75-hTERT increased cell proliferation by 1.25 fold or more in 12 and 21 of 22 cell lines, respectively (Figure 1).

When proliferation assays were performed using culture supernatant obtained from fibroblasts established from normal esophagus (HEF 2111) and purchased lung-derived fibroblasts (HFL-III), generally similar trends are observed (data not shown). These findings imply that growth factors or cytokines secreted by fibroblasts play an important role in cancer cell proliferation.

### Gene expression

**Figure 2:**
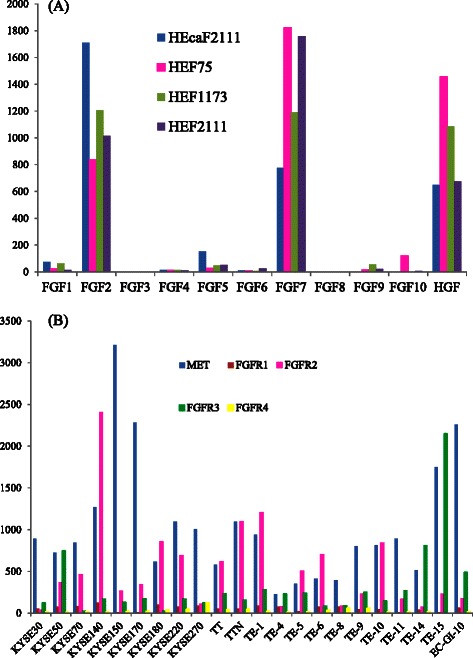
**Gene expression data of the components of the HGF/MET and FGF/FGFR systems in esophageal fibroblasts and ESCC cell lines. (A)** The gene expression of HGF and FGF1-10 in esophageal fibroblasts is shown. High expression of FGF2 and FGF7 is consistently found in four types of esophageal fibroblasts, three derived from normal esophagus (HEF75, HEF1173, and HEF2111) and one derived from tumor (HEcaF2111). The bar graph shows the expression levels of the indicated genes. **(B)** The gene expression levels of MET and FGFR1-4 in 22 ESCC cell lines. MET and FGFR2 are highly expressed in the majority of ESCC cell lines. The bar graph shows the expression levels of the indicated genes. The expression data were normalized with the Affymetrix MAS5.0 algorithm with target intensity of 100.

We hypothesized that HGF and FGFs secreted by fibroblasts may be responsible for the stimulatory effect of fibroblast supernatant [13,23,24,27,40]. To test this hypothesis, we studied gene expression profile data to determine if fibroblasts and ESCC cells expressed the ligands (HGF and FGFs) and their receptors (MET and FGFR1-4). The relevant results are shown in Figure 2. Relatively high expression levels of FGF2, FGF7 and HGF are observed with fibroblasts established from tumor (HECAF2111) and three types of fibroblasts established from normal tissue (HFF75, HEF1173, and HEF2111). Analysis of the cancer cell lines shows a variable, but widespread expression of MET and FGFR2, which are receptors for HGF and FGF7, respectively, across the panel of ESCC cell lines (Figure 2).

### Extrinsic and intrinsic roles of HGF/MET and FGFs/FGFR in ESCC cell proliferation

**Figure 3:**
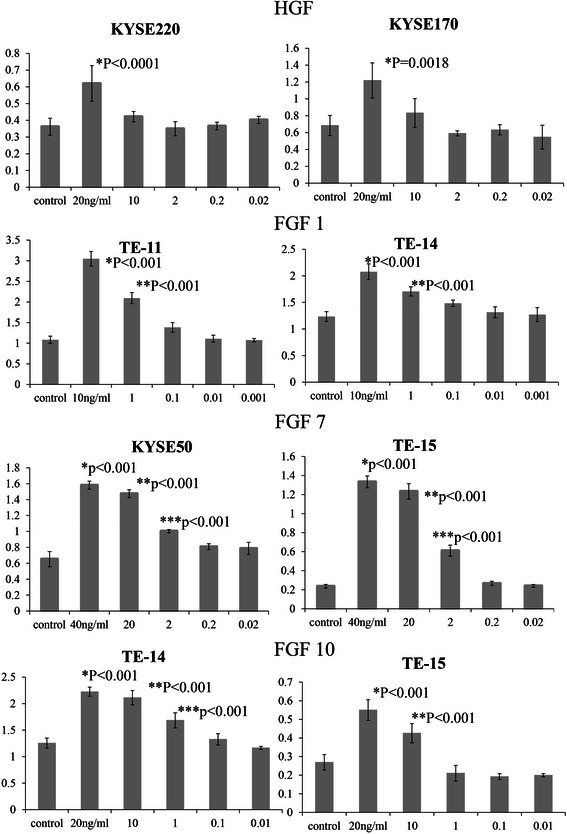
**Changes in the proliferation of esophageal cancer cell lines after addition of HGF and FGF1, 7, and 10.** Data for representative cell lines are shown as bar graphs. Proliferation is significantly induced by HGF in KYSE220 and KYSE170 cell lines. Significant proliferation of TE-11 and TE-14 is induced by FGF1 at a concentration of 1 ng/mL or higher. Significant proliferation of KYSE50 and TE-15 is induced by FGF7 at a concentration of 2 ng/mL or higher. For each cell line, the P-value is less than 0.001 on one-way analysis of variance. Dunnet’s test was used for comparison with the control group, and P-values were calculated. The Y axis indicates the absorbance.

**Figure 4:**
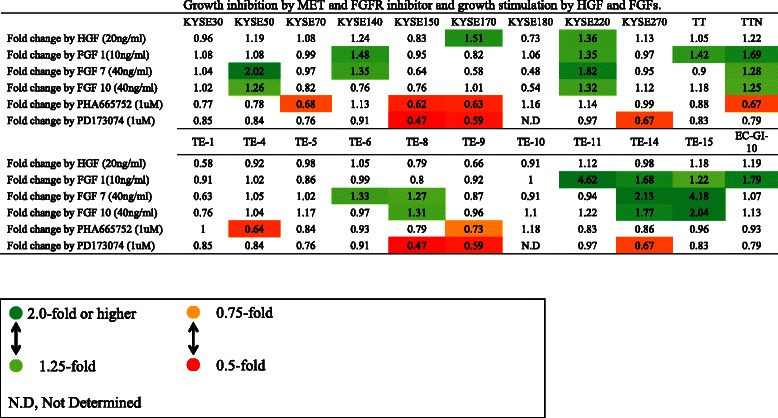
**Growth stimulation by HGF and FGFs and growth inhibition by MET inhibitor and FGFR inhibitor.** HGF stimulates the proliferation of two cell lines (KYSE170 and KYSE220) by more than 1.36 fold, while the addition of FGFs (1, 7, and 10) induces a 1.25-fold or higher growth stimulation in 11 cell lines. MET inhibitor (PHA-665752) inhibits proliferation by 25% or more in six cell lines. FGFR inhibitor (PD-173074) inhibits proliferation by 25% or more in six cell lines, three of which are also growth inhibited by MET inhibitor.

Next, we examined the effects of adding growth factors HGF, FGF1, FGF7, and FGF10, to the culture medium of cancer cells. These growth factors, assumed to be present in the culture supernatants of fibroblasts, may exert a paracrine effect on the proliferation of cancer cells [23,24,27,40]. The results are shown in Figures 3 and 4. HGF stimulates the proliferation of two cell lines (KYSE170 and KYSE220) by more than 1.36 fold, but the stimulatory response is less marked in the other 20 cell lines. FGF1, FGF7, and FGF10 induce the proliferation of four, four, and two cell lines by 1.5 fold or more, respectively. In particular, FGF7 induces the proliferation of eight cell lines by a factor of 1.25 fold or more, and overall, FGFs stimulated the proliferation of 11 cell lines by 1.25 or more. We also examined the intrinsic role of HGF/MET and FGFs/FGFR in ESCC proliferation. Cell proliferation assays were performed after adding 0.1% of DMSO (control), PD-173074 (MET inhibitor), or PD-173074 (FGFR inhibitor). As compared with control, PHA-665752 and PD-173074 each inhibit cell proliferation by 25% or more in six cell lines (Figure 4).

These results indicate that cell proliferation of many ESCC cell lines is dependent on HGF/MET and FGF/FGFR either in an extrinsic or intrinsic manner. These experiments also indicate diversity in the degree of dependence on HGF/MET and FGF/FGFR among the cell lines tested, with more cell lines exogenously dependent on FGF/FGFR than on HGF/MET.

### Western blot analysis

Both Akt and ERK1/2 participate in the biological effects of growth factors [41-43]. The ERK signaling cascade is reported to control the proliferation of multiple cell types in response to growth factor treatment [44,45]. In contrast, Akt signaling is best known for mediating cell survival [43].

**Figure 5:**
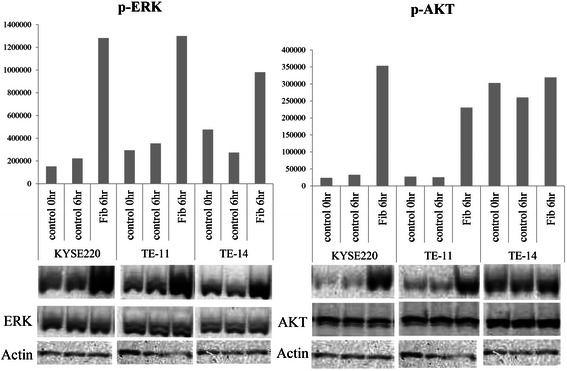
**Changes in the phosphorylation of ERK and AKT after adding fibroblast (HEF -75hTERT) culture supernatant.** In representative cell lines (TE-11, TE-14, and KYSE220), we examined the phosphorylation of ERK and AKT six-hours after the addition of supernatant. Both p-ERK and p-AKT levels are markedly up-regulated by treatment with fibroblast supernatant, though neither total AKT nor total ERK are changed. The Y axis indicates the pixel/area.

Cell lines with proliferation induced by fibroblast supernatants (TE-11, TE-14, and KYSE220) were selected. Western blot analysis was performed to assess the phosphorylation of AKT and ERK1/2 6 hours after adding supernatant. As shown in Figure 5, treatment with fibroblast supernatant markedly up-regulates p-ERK protein levels in all three cell lines tested (Figure 5). p-AKT protein levels are also clearly up-regulated by supernatants in two cell lines (KYSE220 and TE-11), and slightly upregulated in one cell line (TE-14).

### Effects of PHA-665752 (MET inhibitor) and PD-173074 (FGFR inhibitor) on cell proliferation induced by fibroblast supernatant

**Figure 6:**
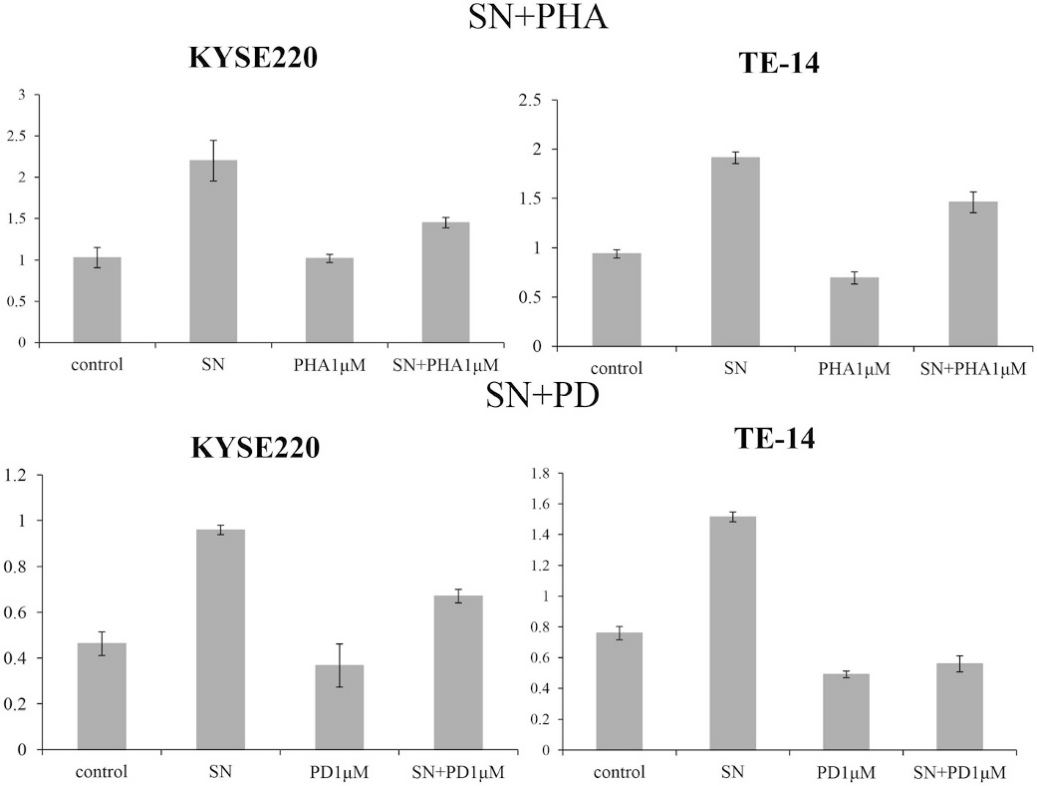
**Effects of a MET inhibitor (PHA-665752) and an FGFR inhibitor (PD-173074) on cell proliferation induced by the addition of fibroblast culture supernatant.** After the addition of 1 μM PD-173074 or 1 μM PHA-665752 to fibroblast (HEF75-hTERT) culture supernatant, cell proliferation assays were performed. The results for representative cell lines are shown as bar graphs. The Y axis indicates the absorbance.

To investigate the relative contribution of HGF/MET and FGFs/FGFR to the stimulatory effect of fibroblast supernatant, we tested the effect of PD-173074 (FGFR inhibitor) and PHA-665752 (MET inhibitor) treatment on cell proliferation induced by fibroblast supernatant. The results are shown in Figure 6. When the MET (HGF receptor) inhibitor PHA-665752 was added to fibroblast culture supernatants at a final concentration of 1 μM, stimulation by fibroblast supernatant is modestly blunted in KYSE220, but not significantly affected in TE-14. In contrast, when the FGFR inhibitor (PD-173074) is added to fibroblast culture supernatants at a final concentration of 1 μM, the opposite pattern is observed. Stimulation by fibroblast supernatant is markedly blunted by FGFR inhibitor in TE-14, but the blunting effect of FGFR inhibitor is less obvious in KYSE220. We calculated the blunting index for each inhibitor, as described in the Methods section. A high blunting index for PHA-665752 indicates that the stimulating effect of fibroblast supernatant is dependent on HGF/MET. The blunting index for PHA-665752 was 34.15 and 23.60 in KYSE220 and TE-14, respectively. The blunting index for PD-173074 was 30.30 and 63.05 in KYSE220 and TE-14, respectively (Figure 6). These results suggest that stimulation by fibroblasts is more dependent on HGF/MET than on FGFs/FGFR in KYSE220, while it is dependent more on FGF/FGFR than on HGF/MET in TE-14.

Testing all ESCC cell lines for the relative contribution of HGF/MET and FGF/FGFR in a similar experiment, demonstrates that four cell lines (KYSE220, TTN, TE-6, and TE-15) are dependent on HGF/MET, while eight cell lines (KYSE30, KYSE50, TT, TE-6, TE-9, TE-11, TE-14, and TE-15) are dependent on FGF/FGFR. These results indicate that paracrine stimulation by fibroblast is dependent, at least in part, on HGF/MET or FGF/FGFR. These results also show that there is diversity among ESCC cell lines with regard to dependence on HGF/MET and FGF/FGFR.

### Inhibitory effects of lapatinib on ESCC cell proliferation and rescue by fibroblast supernatant

Cancer treatment strategies are currently shifting to targeted therapies. Several drugs are target members of the human epidermal growth factor receptor (EGFR/HER) family [46]. This family includes four membrane receptor tyrosine kinases, EGFR (HER1/erbB1), HER2 (erbB2), HER3 (erbB3), and HER4 (erbB4), which activate key cell signaling pathways controlling cell growth, proliferation, migration, apoptosis, and resistance to cytotoxic agents [47]. Wang et al. reported that supernatants of human fibroblast cell lines induce gefinitib resistance in lung cancer cell lines [48].

**Figure 7:**
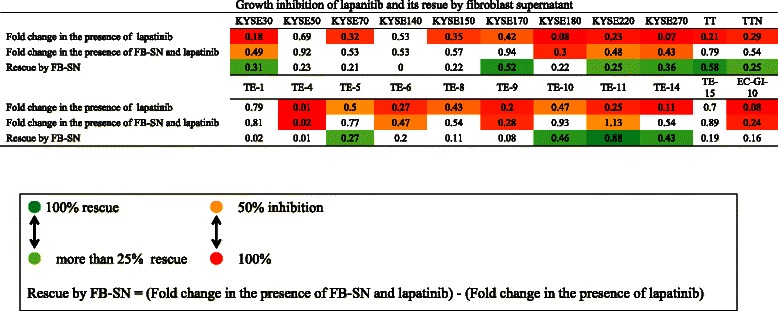
**The inhibitory effect of lapatinib on the proliferation of 22 cancer cell lines and the rescue effect of fibroblast culture supernatant against lapatinib-induced inhibition.** Lapanitib (1 μM) inhibits cell proliferation by more than 50% in the majority of the ESCC cell lines. Addition of fibroblast supernatant rescues growth inhibition by lapatinib and recovers cell proliferation by 20% or more in 10 cell lines.

In this study we examined the inhibitory effect of lapatinib on ESCC cell lines. In addition, we studied whether the fibroblast supernatants affect the inhibitory effect of lapatinib on ESCC cell proliferation. The results are shown in Figure 7. Lapanitib at 1 μM inhibits cell proliferation by more than 50% in the majority of the ESCC cell lines. Addition of fibroblast supernatant rescues growth inhibition by lapatinib and recovers cell proliferation by 20% or more in 10 cell lines. These findings suggest that fibroblast supernatants might induce resistance to lapatinib in ESCC cell lines.

### FGFR inhibitor (PD-173074) blocks the rescue effect of fibroblast supernatants against lapatinib

Recent studies indicate that paracrine stimuli from stromal fibroblasts may confer resistance to various molecular targeted agents including lapatinib [49-51]. We reasoned that blockade of paracrine stimuli may attenuate the rescue effect of fibroblast supernatant in lapatinib-sensitive ESCC cell lines. We chose TE-11 and TE-14 cell lines in which the paracrine effect of fibroblasts are dependent on FGFRs, and tested the effect of FGFR inhibitor on the rescue effect of fibroblast supernatant.

**Figure 8:**
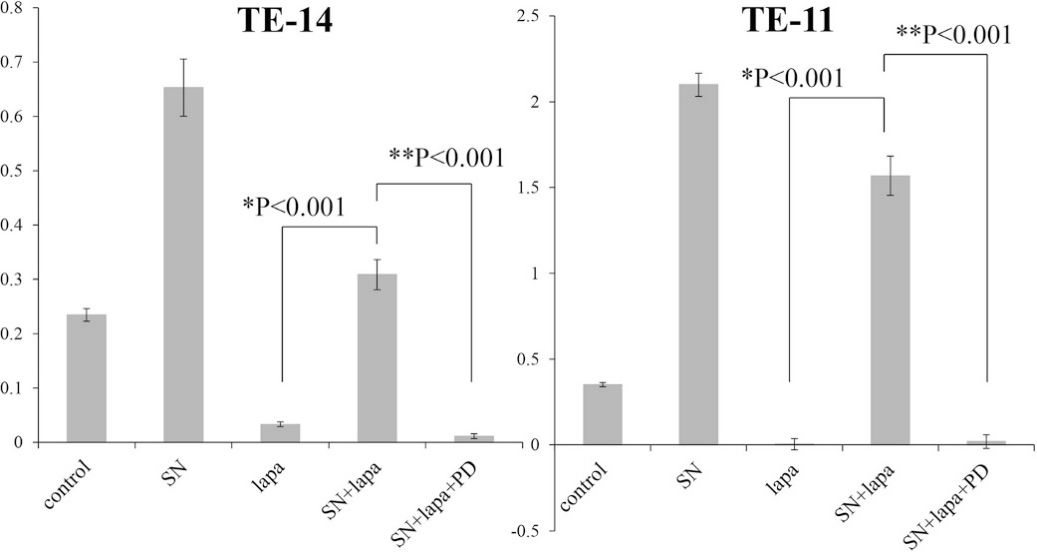
**Rescue from lapatinib inhibition by FB-SN and abrogation by FGFR inhibitor.** The inhibitory effect of lapatinib on the proliferation of representative cell lines is rescued by the addition of fibroblast culture supernatant. Further addition of FGFR inhibitor (PD-173074) abrogates the rescue effect of fibroblast supernatant. The Y axis indicates the absorbance.

The results of these experiments are shown in Figure 8. Based on a cell count assay, when representative cell lines were additionally treated with 1 μM of FGFR inhibitor (PD-173074), together with fibroblast supernatants and 1 μM of lapatinib, proliferation rates are similar to those obtained after adding only lapatinib to the cancer cells. These results show that PD-173074 blocks the rescue effect of fibroblast supernatants against lapatinib.

### ELISA

The concentrations of HGF in culture supernatants of the fibroblasts HEF75, HEF75-hTERT, and HFL-III were 4049 pg/mL, 9390 pg/mL, and 9381 pg/mL, respectively, and the concentrations of FGF7 were 249 pg/mL, 1087 pg/mL, and 17 pg/mL, respectively. Culture supernatants of HFL-III, a lung-derived fibroblast line, contained high concentrations of HGF, but very low concentrations of FGF7. The culture supernatants of measured cancer cells (KYSE30, KYSE50, KYSE150, KYSE220, T.TN, TE-8, TE-11, TE-14, and TE-15) contained virtually no HGF or FGF7, suggesting that the autocrine activity of these cells was minimal.

## Discussion

The importance of the tumor microenvironment for tumor growth has been recognized for many years [51]. Cancers develop in a complex and dynamic microenvironment. Stromal fibroblasts are one of the major stromal components and it is becoming increasingly clear that fibroblasts are also prominent modifiers of cancer progression [21].

To date, few studies have comprehensively evaluated the interactions between ESCC and stromal fibroblasts. We studied 22 ESCC cell lines, the maximum available commercially, because many previous studies used few cell lines that were appropriate for the experiments. Furthermore, we isolated esophageal stromal fibroblasts from resected specimens and immortalized a portion of them. These studies clarify the roles of fibroblasts in the proliferation of ESCC and in resistance to lapatinib, given the diversity of cancer.

We first examined cell proliferation induced by fibroblasts. The addition of fibroblast supernatant induced cell proliferation in the majority of ESCC cell lines. In particular, supernatants from immortalized fibroblasts had a stronger impact on cancer cell lines. HEF75-hTERT, immortalized fibroblasts, increased cell proliferation by 1.25 fold or more in 21 of 22 cell lines, while normal fibroblasts increased proliferation in 12 of 22 cell lines (Figure 1). These results imply that immortalized fibroblasts may develop properties similar to the so-called cancer associated fibroblasts.

Stromal fibroblasts promote tumor progression in several ways such as secretion of multiple factors and matrix metalloproteinases [52]. We hypothesized that some growth factors, such as HGF or FGFs secreted by stromal fibroblasts may be responsible for the proliferation effect of ESCC cells. In fact, previous studies have suggested that HGF promotes invasion of ESCC cells [23] and FGF7 (keratinocyte growth factor: KGF) increases the growth rate of esophageal cancer cell lines (TE-8 and TE-11) [24]. We next examined the effects of adding growth factors to the culture medium of cancer cells and the effects of FGFR inhibitor and MET inhibitor treatment on cell proliferation induced by fibroblast supernatant. The results of experiments to evaluate the effects of adding growth factors and kinase inhibitors suggest that the stimulating effect of fibroblasts was attributable in part to HGF/MET or FGF/FGFR (Figure 4). The results indicate diversity in the degree of dependence on HGF/MET and FGF/FGFR among the cell lines. The exogenous proliferation of more ESCC cell lines may depend on FGF/FGFR signaling rather than on HGF/MET signaling.

The development of targeted therapies is a major advance in the treatment of cancer [46], but data for esophageal cancer are still lacking [53]. Previous reports imply that targeted therapy has potential for the treatment of patients with ESCC [33]. Stromal fibroblasts also might correlate with resistance to targeted therapy [49,51]. Lapatinib, which is a dual tyrosine kinase inhibitor, is considered a promising candidate [54]. When we added 1 μM of lapatinib to the 22 ESCC cell lines tested, proliferation was strongly inhibited in a majority of the cells lines (Figure 7). However, lapatinib-induced inhibition was abrogated by fibroblast supernatants in many cell lines, and proliferation in 10 of the 22 cell lines was rescued more than 25% (Figure 7). These findings suggest that lapatinib inhibits the proliferation of ESCC cells in an intrinsic manner, while fibroblasts might antagonize the effect of lapatinib. Moreover, these results imply that the effect of targeted therapies can be attenuated by stromal fibroblasts. Fibroblasts might thus have a role in drug resistance, even in ESCC cell lines.

Finally, we tested whether the FGFR inhibitor PD-173074 could eliminate the rescue effect against lapatinib that was induced by fibroblast supernatants. In two representative cell lines (TE-11 and TE-14), fibroblast supernatant restored the growth of cancer cells inhibited by lapatinib, but the subsequent addition of PD-173074 abrogated the rescue effect of fibroblast supernatant against lapatinib (Figure 8). These findings imply that combination therapy with lapatinib and an FGFR inhibitor might be effective in overcoming therapeutic resistance against to lapatinib caused by stromal fibroblasts.

## Conclusions

We studied 22 ESCC cell lines taking into account the diversity of malignancies. In more than half of the cancer cell lines tested, proliferation was induced by culture supernatants of immortalized fibroblasts. Growth factors, such as HGF or FGFs secreted by fibroblasts influence cell proliferation and resistance to lapatinib in some cancer cell lines, although the degree of dependence differs among the cell lines. The addition of fibroblast supernatant attenuates the effect of lapatinib. However, the rescue effect of fibroblast supernatant is abrogated by combining an FGFR inhibitor with lapatinib. Although this study was performed in vitro, these results may lead to the development of new treatment strategies.
